# Investigation of a real-time location system of corneal astigmatic axis

**DOI:** 10.1186/s40662-017-0086-6

**Published:** 2017-09-14

**Authors:** Jian-Guo Zhao, An-Peng Pan, Ke Zheng, A-Yong Yu

**Affiliations:** The Eye Hospital of Wenzhou Medical University, 270 Xueyuan West Road, Wenzhou, (325000) Zhejiang People’s Republic of China

**Keywords:** Corneal astigmatism, Location, Real time, Mark

## Abstract

**Background:**

To construct a real-time computerized location system (RCLS) to analyze and display the axis of corneal astigmatism and to compare its accuracy with the Scheimpflug method.

**Methods:**

Fifty-seven eyes of 39 volunteers with corneal astigmatism more than 1.00 diopter (D) were recruited. The RCLS was composed of a circular light-emitting diode (LED) light source, surgical microscope, surgical video system, computer and self-programming image analysis software. Scheimpflug imaging measurements (Pentacam HR, Oculus, Wetzlar, Germany) were performed on all subjects to determine the axis and power of corneal astigmatism. Thereafter, the axis of corneal astigmatism was analyzed in real-time and displayed by the RCLS on supine position, and videos were recorded. The MB-Ruler 4.0 software was used to measure the astigmatic axis. The accuracy of the RCLS was compared with the Scheimpflug method.

**Results:**

The RCLS was able to display the axis of corneal astigmatism in real-time. The axial deviation of corneal astigmatism between the two methods was 0.63 ± 3.78° when astigmatism was 1.00 to 2.00 D and decreased to 0.06 ± 1.38° when astigmatism was greater than 2.00 D. A linear correlation of astigmatic axis was noted between the two methods: Axis_RCLS_ = 1.01 × Axis_Scheimpflug_ − 1.02 (R^2^ = 0.998, *P* < 0.001). The Bland-Altman analysis revealed that the RCLS agreed sufficiently well with the Scheimpflug method.

**Conclusions:**

The RCLS can accurately analyze and display the axis for corneal astigmatism greater than 1.00 D in real-time. The RCLS simplifies marking procedures and may have potential clinical application to improve the postoperative visual outcomes in surgical correction of corneal astigmatism.

## Background

Correction of pre-existing corneal astigmatism during cataract surgery is increasingly accepted in clinical practice because it can improve visual quality and minimize postoperative spectacles dependence. It had been reported that 30% or more cataract patients have corneal astigmatism greater than 1.00 diopter (D) [1–3], and ocular residual astigmatism may influence postoperative outcomes [4]. Clinically, several methods are available to correct corneal astigmatism during cataract surgery: (1) clear corneal incision at the steep corneal meridian; (2) astigmatic keratotomy at the steep corneal meridian; [5, 6] (3) limbal or peripheral corneal relaxing incision at the steep corneal meridian; [7–9] and (4) implantation of a toric intraocular lens (IOL) [10–15].

For all the surgical methods, accurately locating the axis of pre-existing corneal astigmatism plays an important role. In the clinic, there are four methods commonly used to measure the axis of corneal astigmatism preoperatively: manual-keratometry, optical biometry, auto-keratometry, and the Scheimpflug method. A study carried out by Minwook Chang et al. showed that there were no significant differences among these four methods in measuring the axis of pre-existing corneal astigmatism when correcting astigmatism with toric IOLs [16].

The change between upright and supine position will cause eye cyclotorsion [17], which may bring in possible misalignment between the axis of toric IOLs and corneal astigmatism. To eliminate the off-axis deviation when implanted toric IOLs, the traditional method was to mark reference points at 3- and 9-o’clock on the limbus or conjunctiva under the slit lamp microscope before surgery. Then, based on the two reference points, a protractor was used to mark the desired axis of placement of the IOL. The traditional method, depending on patient cooperation and subjective judgment of different surgeons, was tedious and time-consuming, which increased the probability of off-axis error in surgical correction of astigmatism.

With the high demands on refractive cataract surgery, there is increasing concern about the real-time location of corneal astigmatism, simplifying the marking procedures and improving the accuracy of astigmatism correction. In this study, we built a real-time computerized location system (RCLS) to display the axis of corneal astigmatism, and compared its accuracy with the Scheimpflug method.

## Methods

The volunteers with regular corneal astigmatism of 1.00 D or greater were enrolled in this prospective controlled trial at the Eye Hospital of Wenzhou Medical University, China. Exclusion criteria included any corneal diseases, dry eye whose breakup time of tear film was less than 5 s, active ocular inflammation, contact lens wear within three months, or history of ocular surgery or trauma. This study followed the tenets of the Declaration of Helsinki. All subjects provided informed consent and approval was obtained from the Institutional Review Board of the Eye Hospital of Wenzhou Medical University.

### System construction

**Fig. 1:**
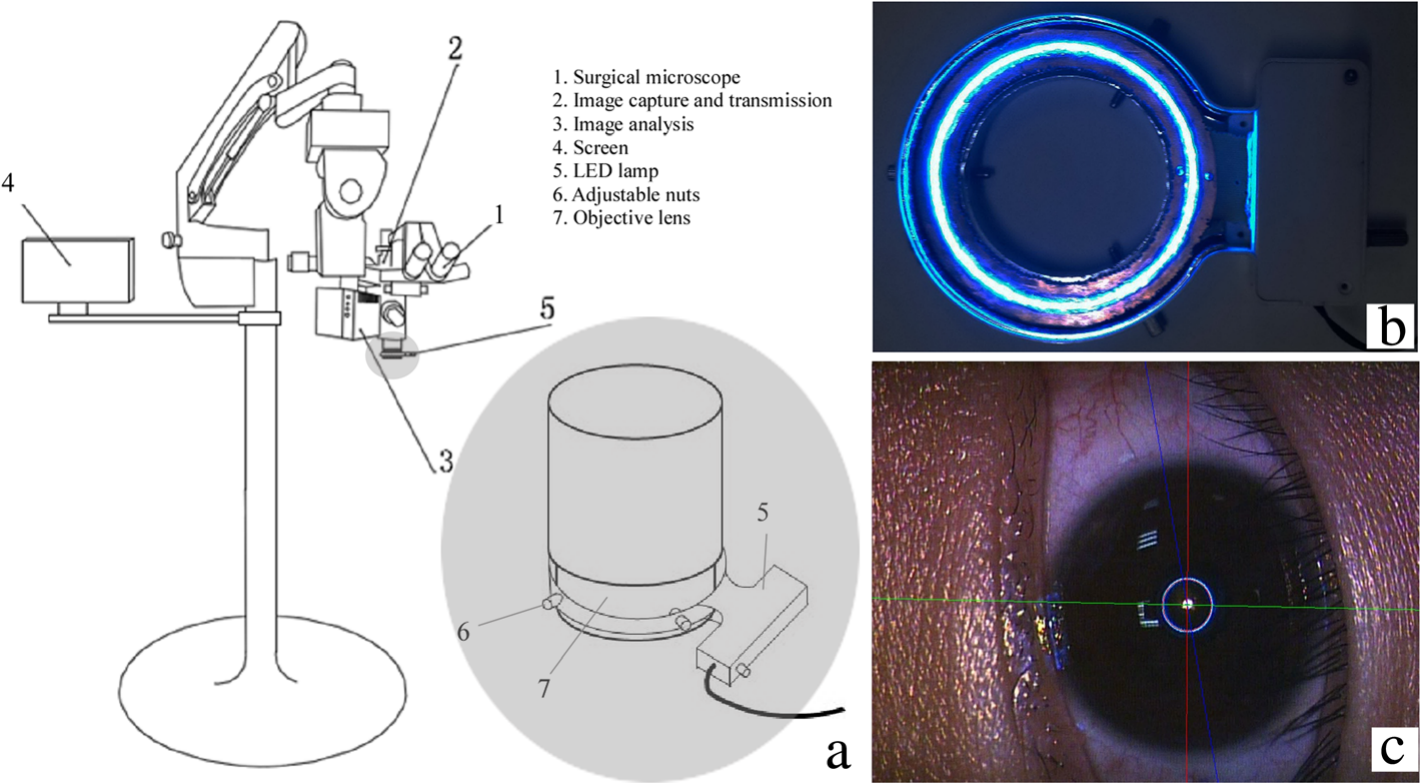
The construction and display interface of Real-time Computerized Location System (RCLS). **a** Schematic diagram of RCLS. **b** LED light source used by RCLS. **c** A real-time case of RCLS: displayed the flat axis of corneal astigmatism (1, red line), the axis of surgical incision (2, blue line), and the pre-determined axis of a toric IOL (3, green line) intraoperatively

The construction of the system has been described in detail in the patent RCLS [18]. The RCLS was mainly composed of a circular light-emitting diode (LED) light source, computer and self-programming image analysis software (Fig. 1a, b). Although the entire cornea was not a sphere, its central 3 mm area could be regarded as a convex mirror [19]. If corneal astigmatism existed, the reflex image of the LED ring on the cornea would be an ellipse, and the major axis of the ellipse could be turned into the axis of corneal astigmatism when analyzed by the software. A perfect circle was used to calibrate the image recording and analysis system before the test. The software analyzed the reflex image of LED circle from the cornea in real-time, and displayed a line on the cornea, which represented the axis of corneal astigmatism. The axis of surgical incision was determined by the surgeon. The pre-determined axis of a toric IOL was calculated by AcrySof Toric Calculators (http://www.acrysoftoriccalculator.com/aspheric/Calculator.aspx). According to the relative angles to the axis of corneal astigmatism input preoperatively, the pre-determined axis of a toric IOL, the axis of the surgical incision, or any other axes could be entered into the software and displayed simultaneously or separately. Figure 1c shows the real-time RCLS simultaneously displaying the flat axis of corneal astigmatism, the pre-determined axis of a toric IOL, and the axis of the surgical incision.

### Axis-marking procedure

All procedures were conducted by the same skilled ophthalmologist. Scheimpflug imaging measurements (Pentacam HR, Oculus, Wetzlar, Germany) were performed on all subjects to measure the axis and power of anterior corneal astigmatism. After topical anesthesia with one drop of 0.5% hydrochloric acid proparacaine (Alcaine, Alcon, USA), subjects were seated in front of a slit lamp microscope and they gazed at a distant target. A narrow slit beam was oriented horizontally and centered on the pupil. The corneal limbus was marked at 3- and 9- o’clock using a disposable medical sterile pen. The MB-Ruler 4.0 software was used to measure the astigmatic axis relative to the 3- and 9- o’clock reference points.

The subjects were asked to lay down on the surgical bed told to gaze directly at the center of the light source of the surgical microscope that was coaxial with the RCLS, which was used to analyze and display the desired axis. The RCLS was performed on each subject and three fragments of the video were recorded. In each fragment, one frame in which the light of microscope was at the center of the cornea was chosen to be analyzed. The MB-Ruler 4.0 software was used to measure the astigmatic axis in degrees in the chosen frames. When the MB-Ruler 4.0 was used to align the 0 scale line to the 3- and 9- o’clock direction, another scale line was the value of astigmatic axis. The average result of three RCLS measurements was used to compare with the Scheimpflug method to analyze the accuracy of axis measurements.

### Statistical analysis

Statistical analysis was performed using MedCalc for Windows (Version 15.11.0; Ostend, Belgium). The relationship of the astigmatic axis between the RCLS and Scheimpflug method was analyzed using the Spearman correlation test. Independent sample t test was performed for comparing average deviation between the low and high astigmatism groups. The Bland-Altman method was used to analyze the agreement between the two methods. In order to assess the correlation and agreement between the two methods, six astigmatic axes were converted accordingly by adding 180° e.g., the correlation and agreement were analyzed between 185° (RCLS) and 180° (Scheimpflug method), rather than 5° (RCLS) and 180° (Scheimpflug method). The level of significance was set at *P* < 0.05.

## Results

This study included 57 eyes of 39 subjects, 18 males (25 eyes) and 21 females (32 eyes). The average age was 37.2 ± 18.8 years old, ranging from 20 to 78 years old. Corneal astigmatism was between 1.00 D to 3.68 D.

**Fig. 2:**
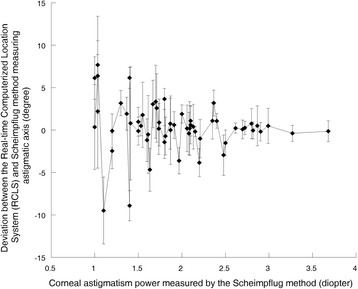
The relationship between the deviation of the astigmatic axis and cylindrical power. The deviation of the astigmatic axis between the Real-time Computerized Location System and Scheimpflug imaging method decreased when cylindrical power increased (*P* = 0.004)

Table 1 and Fig. 2 show the deviation of the astigmatic axis between the RCLS and Scheimpflug method. The deviation decreased significantly when cylindrical power was increased.

**Table 1:** Deviation of corneal astigmatic axis between the Real-time Computerized Location System (RCLS) and Scheimpflug imaging method

Cylindrical power (Diopters)	Axial deviation (Degrees)	*P* value
1.00 to 2.00	0.63 ± 3.78	0.004
>2.00	0.06 ± 1.38

**Fig. 3:**
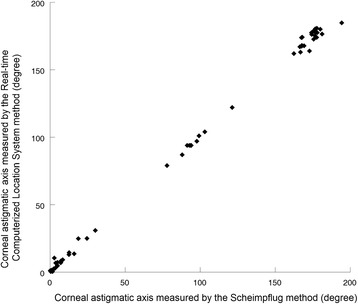
Correlation between the Real-time Computerized Location System and Scheimpflug imaging system in measuring corneal astigmatic axis: Axis_RCLS_ = 1.01 × Axis_Scheimpflug_ − 1.02 (R^2^ = 0.998, *P* < 0.001)

There was a linear correlation of corneal astigmatic axis between the RCLS and Scheimpflug method: Axis_RCLS_ = 1.01 × Axis_Scheimpflug_ − 1.02 (R^2^ = 0.998, *P* < 0.001, Fig. 3).

**Fig. 4:**
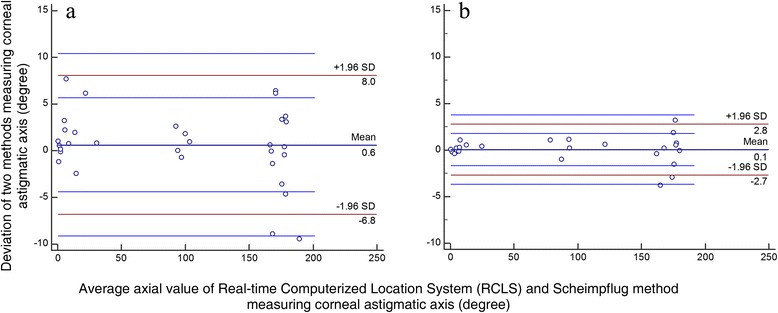
Bland-Altman analysis. The dotted lines represent 95% Limits of Agreement (LoA). The intermediate solid line represents the mean of the deviation. The shorter four solid lines represent the 95% Confidence Interval (CI) of LoA. **a** When corneal astigmatism was 1.00 to 2.00 D, the CI of LoA was −9.14° to 10.40°, 1 point (1/32, 3.1%) outside the CI of LoA (*P* = 0.35). Within the agreed limits, the maximum and average absolute axial deviation of corneal astigmatism between the Real-time Computerized Location System (RCLS) and Scheimpflug method were 8.91° and 0.63°; **b** When corneal astigmatism was greater than 2.00 D, the CI of LoA was −3.64° to 3.77°, 1 point (1/25, 4.0%) outside the CI of LoA (*P* = 0.82). Within the agreed limits, the maximum and average axial deviation of corneal astigmatism between RCLS and Scheimpflug method were 3.19° and 0.06°

According to the Bland-Altman analysis (Fig. 4), when astigmatism was 1.00 to 2.00 D, there were 96.9% of points within the Confidence Interval (CI) of Limits of Agreement (LoA) (*P* = 0.35), in which the maximum absolute axial deviation was 8.91°. The axial deviation outside the CI of LoA was −9.47°. When astigmatism was greater than 2.00 D, there were 96.0% of points within the CI of LoA (*P* = 0.82), in which the maximum absolute axial deviation was 3.19°. The axial deviation outside the CI of LoA was −3.83°.

## Discussion

Accurate localization of corneal astigmatic axis plays a significant role in the successful correction of pre-existing astigmatism. This study constructed the RCLS to display the corneal astigmatic axis in real-time, and the results were found to agree sufficiently well with the Scheimpflug method when corneal astigmatism is greater than 1.00 D. The Bland-Altman analysis revealed that when astigmatism was 1.00 to 2.00 D, the maximum and average absolute axial deviation in the CI of LoA between the RCLS and Scheimpflug method were 8.91° and 0.63°. There was only one point outside the CI of LoA. When astigmatism was greater than 2.00 D, the maximum and average absolute axial deviation in the CI of LoA between the RCLS and Scheimpflug method was 3.19° and 0.06°. There was only one point (2.20 D, −3.83°) outside the CI of LoA. Thus, we concluded that when astigmatism is greater than 1.00 D, the RCLS and Scheimpflug method could be interchangeable in the clinic in terms of locating corneal astigmatic axis. In fact, the RCLS became more accurate with increasing astigmatism.

The RCLS can detect and display the axis of corneal astigmatism in real-time by analyzing the corneal reflex image. Location of the axis of corneal astigmatism is one of the hot topics in the field of toric IOL implantation. A new location method has been introduced by Cha et al. [20] for toric IOL implantation. In Cha’s method, an anterior segment photograph was taken to identify reference vessels and marking points. Actual distance was calculated from reference vessel points to axis marking points using an expression derived from the sizes of the photographed image and the actual cornea. Finally, the axis marking points were marked on the limbus during surgery. Cha’s axis-marking method simplified the steps and was a good alternative for those with sunken eyes or with poor cooperation. However, there were several factors that should be taken into consideration: 1) the reference vessel points must not be far away from the corneal limbus; 2) the method was unable to identify the axis for those lacking limbal vessels or vessels in color that were too slight to be recognized; 3) it may take the surgeon some time to familiarize himself with the photoshop program used preoperatively; 4) minimal scale calipers used may produce errors.

In traditional axis-marking methods, preoperative marking of reference points and the stability of toric IOLs were the most significant surgical factors to determine the postoperative visual outcomes in high astigmatism. With the development of material and design of toric IOLs, significant rotation of IOLs rarely occurred postoperatively. Mencucci et al. reported that the mean IOL rotation was 3.00 ± 1.69° postoperatively [21]. Chassain C provided a similar result that the mean IOL rotation was 2.16 ± 1.95° [22]. According to Visser N et al., a commonly used 3-step ink-marker procedure before implanting toric IOLs produced a mean off-axis error of approximately 5° [23]. If the error of preoperative marking and the rotation of an implanted IOL both rotated toward the same clockwise, a total off-axis error of a toric IOL to the pre-determined axis will be up to approximately 10° and may lead to a significant loss of astigmatic correction. The RCLS simplified the marking procedures and did not need any additional steps before the patient laid down on the surgical bed. What a surgeon only needed to do was to check the parameters entered in the software, thus eliminating the off-axis error arising from preoperative horizontal marking as used in traditional marking method.

The RCLS can also display in real-time the axis of surgical incision based on the relative angle to the axis of corneal astigmatism if it was calculated and input at the beginning of surgery. In the implantation of toric IOLs, precise corneal incision positioning is another important factor to guarantee the efficacy of surgical correction of corneal astigmatism. Studies had shown that a 2.2 mm incision produced 0.31 ± 0.54 D surgically induced astigmatism (SIA), and a 2.75 mm incision produced 0.56 ± 0.42 D SIA [24]. Thus, the surgical incision should be calculated and placed at the certain pre-determined axis, otherwise, it will lead to new astigmatism. Traditional marking methods took several steps to mark the pre-operative reference points on the corneal limbus, and the incision location was determined based on its relative position to the reference marking, which, again, was time-consuming and increased the probability of off-axis error. In addition, the minimum scale of the axis marker was 5° to 10°, which further highlighted its inability to precisely mark the axis. Therefore, the simultaneous display of the axis of the surgical incision in the RCLS can be a potential application to improving the efficacy of surgical correction of corneal astigmatism.

Currently, there are several real-time imaging technologies available for intraoperative toric IOL alignment. These include the VERION Digital Marker (Alcon Laboratories, Ft. Worth, TX), Callisto Eye with Z-Align (Carl Zeiss Meditec AG, Jena, Germany), the iTrace with Zaldivar Toric Caliper (Tracey Technologies, Houston, TX). The key steps for the operation of these systems are: 1) obtaining a high-resolution preoperative image that contains the limbal vessels, iris feature and/or scleral vessels; 2) intraoperative registration of the surgical eye based on iris landmarks, scleral and limbal vessels; and 3) digitally display the intended toric IOL axis intraoperatively for toric IOL alignment. These imaging technologies are real-time ‘display’ systems, rather than a real-time ‘measure’ system, where its purpose is to improve the accuracy of toric IOL alignment. Moreover, in the femtosecond laser–assisted cataract surgery, the vacuum rise during the suction may induce a circumferential subconjunctival hemorrhage, which can interfere with image recognition and eye registration intraoperatively. In addition, the area outside the central cornea, especially the part of the limbus, can be blocked by surgical instruments during surgery, which, again, will interfere with eye registration. In contrast, the RCLS is a real-time ‘measure’ and ‘display’ system as it can analyze and display the astigmatic axis intraoperatively. The RCLS also shows great compatibility even with the femtosecond laser–assisted cataract surgery because it only analyzes the central cornea (unaffected by subconjunctival hemorrhage and/or surgical instruments), and there is no need for image recognition and eye registration. Another intraoperative ‘measure’ system is the Optiwave Refractive Analysis (ORA) System (WaveTec Vision Systems Inc., Aliso Viejo, CA) wavefront aberrometer, which measures aphakic refractive status intraoperatively after lens removal and then calculates the IOL power along with the desired toric IOL axis. However, the astigmatism axis measured after lens removal may be affected by surgical incision and corneal edema, therefore, will be different with the postoperative corneal astigmatism axis after corneal edema subsides and corneal incision healing. Most formulas use the preoperative astigmatism measurement rather than the intraoperative astigmatism measurement after lens removal, to design the intended toric IOL axis (also considered as SIA).

However, the RCLS still has several additional factors to be considered. The accuracy of the RCLS will still be questioned despite the sufficient agreement between RCLS and Scheimpflug method because the test-to-test variability of Scheimpflug method has been a point of concern. [25] Furthermore, the repeatability of the RCLS has not been assessed in this study, further investigation to fully assess the repeatability of the RCLS and comparing its accuracy with a more reliable device is needed. The system can only detect corneal astigmatism of an area within the central 3 mm area. The low resolution of the CCD receiving the corneal reflex image decreased pixel points of the output image for precise analysis. The width of the LED circle, which resulted in a certain width of reflection image, increased the difficulty of image recognition. In future, higher accuracy may be expected with the following improvements: 1) the use of infrared light to reduce a patient’s discomfort; 2) increasing the resolution of the surgical video system. Furthermore, the RCLS will be compared with commercial devices such as the Alcon Wavetec ORA, Verion, and Zeiss Callisto eye.

## Conclusion

In conclusion, the RCLS can accurately analyze and display the astigmatic axis when corneal astigmatism is 1.00 D or greater, and the results agree sufficiently well with the Scheimpflug method. The RCLS analyzes corneal astigmatic axis directly based on the corneal optical feature, rather than the anatomic feature of the non-corneal ocular surface to indirectly locate the axis as used in other methods. Therefore, the RCLS can eliminate the effects of eye cyclotorsion and simplify marking procedures i.e., by using the RCLS, tedious preoperative data storage, intraoperative data transmission and intraoperative eye registration based on the preoperative image can be effectively avoided. The RCLS may be a potential clinical application for improving the postoperative visual outcomes in the surgical correction of corneal astigmatism.
